# A Novel Non-invasive Method to Detect RELM Beta Transcript in Gut Barrier Related Changes During a Gastrointestinal Nematode Infection

**DOI:** 10.3389/fimmu.2019.00445

**Published:** 2019-03-12

**Authors:** Norus Ahmed, Emanuel Heitlinger, Nicole Affinass, Anja A. Kühl, Natasa Xenophontos, Victor Hugo Jarquin, Jenny Jost, Svenja Steinfelder, Susanne Hartmann

**Affiliations:** ^1^Department of Veterinary Medicine, Institute of Immunology, Freie Universität Berlin, Berlin, Germany; ^2^Research Group Ecology and Evolution of Molecular Parasite Host Interactions, Leibniz Institute for Zoo and Wildlife Research, Berlin, Germany; ^3^Institute for Biology, Molecular Parasitology, Humboldt Universität, Berlin, Germany; ^4^Charité–Universitätsmedizin Berlin, Corporate Member of Freie Universität Berlin, Humboldt-Universität zu Berlin, and Berlin Institute of Health, iPATH.Berlin, Berlin, Germany; ^5^Department of Neuroscience, Max Delbrück Center for Molecular Medicine, Berlin, Germany

**Keywords:** intestinal nematode, gut barrier, RELM-β, non-invasive method, feces

## Abstract

Currently, methods for monitoring changes of gut barrier integrity and the associated immune response via non-invasive means are limited. Therefore, we aimed to develop a novel non-invasive technique to investigate immunological host responses representing gut barrier changes in response to infection. We identified the mucous layer on feces from mice to be mainly composed of exfoliated intestinal epithelial cells. Expression of RELM-β, a gene prominently expressed in intestinal nematode infections, was used as an indicator of intestinal cellular barrier changes to infection. RELM-β was detected as early as 6 days post-infection (dpi) in exfoliated epithelial cells. Interestingly, RELM-β expression also mirrored the quality of the immune response, with higher amounts being detectable in a secondary infection and in high dose nematode infection in laboratory mice. This technique was also applicable to captured worm-infected wild house mice. We have therefore developed a novel non-invasive method reflecting gut barrier changes associated with alterations in cellular responses to a gastrointestinal nematode infection.

## Introduction

Soil transmitted, intestinal nematodes affect around 24% of the world's population (1) and are prevalent in wild animals. The majority of parasitic helminths live in the gut and are in close contact with the host's epithelial cell (2), representing an important barrier during infection (3). The gut barrier is composed of specific enterocytes interspersed by goblets cells that secrete the cysteine rich cytokine RELM-β and antimicrobial peptides, together forming the epithelial barrier during infection (4). This barrier defends against pathogen invasion but also leads to the activation of the mucosal immune system in the underlying lamina propria. In helminth infections, the activation of the mucosal immune system leads to the activation of a T helper 2 (Th2) immune response. During a mouse gastrointestinal (GI) infection with *Heligmosomoides polygyrus*, the Th2 immune response is characterized by the expression of the transcription factor GATA-3, the cysteine-rich cytokine resistin-like molecule-beta (RELM-β), the cytokines interleukin (IL)-4, IL-5, IL-9, IL-13 and the antibodies IgE and IgG1 (5–7). This Th2 response leads to reduced worm fecundity and parasite expulsion (8, 9). In this scenario, RELM-β has been shown to play multiple roles in different aspects of host defenses. It aids in spatial separation of the colonic epithelium and the microbiota by acting as a bactericidal protein (10, 11). Additionally, RELM-β plays a role in immune regulation and host defenses against intestinal nematode infections. In a *H. polygyrus* infection it prevents worm feeding on host tissues and contributes to the weep and sweep response (12, 13). Notably, during *H. polygyrus* infection significant changes in the composition of the gut microbiota have been documented (13, 14).

Currently, different diseases are detectable using non-invasive techniques that utilize samples from urine, saliva, and stool. Urine samples can be used to detect selected viral, bacterial and parasitic diseases (15, 16), including typhoid fever (17) and eggs of the blood fluke *Schistosoma haematobium* (18). Saliva has been a useful source in the early detection of foot and mouth disease in wild boar (19). Moreover, bacterial infections, such as *Helicobacter pylori* (20) and the parasite *Plasmodium falciparum* are detected non-invasively using saliva from infected patients (16). Stool is regularly used to monitor numerous wildlife populations, including detection of virus infections in gorillas (21) or wild apes (22), bacterial shedding in the European badger as well as helminth eggs in Asian elephants (23, 24) *or African buffalo*s (25). In addition, stool is also used in humans to detect cytomegalovirus DNA instead of using mucosal biopsies (26).

In gastrointestinal helminth infections various helminth eggs are detectable using stool, however this method does not detect the early stages of infection. Neither does it investigate host parameters associated with disease, such as gut barrier related changes. Therefore, a non-invasive sampling technique that provides further information about cellular changes during infection and enables the monitoring of disease progression is urgently required for both laboratory and field settings. A non-invasive assessment reflecting cellular and immunological gut parameters would provide a better understanding of pathogen burdens, detection of communities prone to diseases and the identification of immunologically naïve populations (15, 27). Previously, it has been described that the gastrointestinal epithelium is frequently renewed and cells are shed daily into the fecal stream. A large number of these exfoliated cells have been shown to be intact and viable (28).

Here, we describe a novel non-invasive method that uses exfoliated intestinal cells to monitor cellular and immunological changes of the gut barrier in response to infection. We establish and demonstrate this method in laboratory mice infected with the small intestinal nematode *H. polygyrus*. By comparing acute vs. chronic infection, dose-dependent responses and reinfection after abrogation of infection, we illustrate the potential of detecting cellular responses after gut barrier changes using RNA extracted from exfoliated intestinal cells. In addition, we applied this method to wild mouse stool samples. Thus, this study uses the gene expression from exfoliated cells present on stool to detect changes in gut barrier function due to infectious diseases by non-invasive means.

## Materials and Methods

### Animals

Female BALB/c mice were used (8 weeks old; purchased from Janvier, Saint Berthevin, France). The experiments performed followed the National Animal Protection Guidelines and were approved by the German Animal Ethics Committee for the protection of animals (G0253/14 and G0113/15).

### Infection

*H. polygyrus* was maintained by serial passage in C57BL/6 mice, described previously (30). Mice aged 8 weeks were infected by oral gavage with either 20 or 200 infective L3 larvae diluted in drinking water. On either 14 or 35 days post infection (dpi) mice were sacrificed by isofluorane inhalation. Mice were treated during the acute phase of infection (day 14 and day 15) with 2 mg/animal pyrantel pamoate for adult worm expulsion (Sigma, St. Louis, MO, USA) in 200 μL water.

### Fecal Collection and Storage

4-10 freshly excreted fecal pellets were collected from mice. Fecal pellets were collected in cryotubes, placed into liquid nitrogen and stored at −80°C until processing.

### Wild Mice Sampling

House mice (*Mus musculus*) were captured in autumn 2017 using live traps (approval number 35−2014–2347). Traps were set overnight in farms and private properties in the state of Brandenburg in Germany. All animals were transferred to individual cages and remained there until fecal samples were collected on the following day. Around 4–5 pellets of fresh fecal samples were kept in liquid nitrogen during transportation and maintained at −80°C until processing. Mice were euthanized, digestive tracts were dissected and helminths were identified and counted under a binocular microscope.

### Oocyst Flotation and Counting

For detection and quantification of *Eimeria*, parts of fecal samples were stored in potassium dichromate solution 2.5% (w/v). Oocyst were flotated using a saturated salt solution (specific gravity = 1.18−1.20), collected by centrifugation (3,234 × *g*/room temperature/ 10 minutes) and washed with distilled water (1,800 × *g*/room temperature/10 min). To estimate the intensity of infection, flotated oocysts were counted using a Neubauer chamber under a Leica® DM750 M light microscope at 10X magnification. Results were expressed in oocyst per gram (OPG) of feces.

### *Eimeria* qPCR

To quantify *Eimeria* in intestinal tissue, DNA from cecum and ileum was isolated using the innuPREP DNA Mini Kit (Analytik Jena AG, Jena, Germany) after disruption of the tissue with liquid nitrogen in a mortar. A short fragment of the mitochondrial cytochrome C-oxidase subunit I (mt COI) of *Eimeria* was amplified in a qPCR using the primers TGTCTATTCACTTGGGCTATTGT (Eim_COI_qX-F) and GGATCACCGTTAAATGAGGCA (Eim_COI_qX-R). Every reaction contained 1X iTaqTM Universal SYBR® Green Supermix (Bio-Rad Laboratories GmbH, München, Germany), 400 nM of each primer and 50 ng of DNA template in final volume of 20 μL. Cycling amplification were carried out in a Mastercycler® RealPlex 2 (Eppendorf, Hamburg, Germany) with the following program: 95°C initial denaturation (2 min) followed by 40 cycles of 95°C denaturation (15 s), 55°C annealing (15 s) and 68°C extension (20 s). Melting curve analysis was performed to detect primer dimer formation and non-specific amplification. The CDC42 gene of the mouse nuclear genome was amplified using primers Ms_gDNA_CDC42_F CTCTCCTCCCCTCTGTCTTG and Ms_gDNA_CDC42_R TCCTTTTGGGTTGAGTTTCC as an internal reference. Relative quantification of *Eimeria* DNA as achieved as the ΔCt between mouse and *Eimeria* (CtMouse–CtEimeria). The threshold for detection was estimated at ΔCt = −5 and results above this value were considered as an estimate of parasite tissue load. If both ΔCtIleum and ΔCtCecum indicated infection the higher value was used as estimate for tissue load.

Fresh fecal samples were kept in liquid nitrogen during transportation and maintained at −80°C until processing. Mice were euthanized, digestive tracts were dissected and helminths were identified and counted under a binocular microscope.

### Intestinal Exfoliated Epithelial Cell Extraction

#### Flotation Method

Falcon tubes (15 mL) were filled with 3 mL PBS and put on ice. Fecal pellets previously stored at −80°C were used and one pellet was placed individually into each falcon tube. Tubes were placed on a rocker for 45 minutes at 4°C. Tubes were then turned upright on ice for a further 20–30 min to allow cells to loosen up. Another set of falcon tubes were prefilled with 3 mL PBS and stored on ice. Individual falcon tubes containing a single fecal pellet were slowly inverted until the layer of epithelial cells started to detach from the fecal pellet (Supplementary Video 1). The inverting force on the falcon tubes was slowly increased until the layer floated off. Epithelial cells could also be removed using a pipette while rotating the fecal pellet to completely peel off the layer. These cells were then collected using a pipette and placed into the freshly prepared falcon tubes to remove any fecal debris. This material was subsequently used for RNA extraction (Analytik Jena, Jena, Germany).

#### Alternative Field Method

Fecal pellets were placed in 2 mL tubes filled with 1 mL RNA later (Sigma-Aldrich, St. Louis, MO, USA) and stored at −20°C.For the removal of exfoliated epithelial cells samples were placed in 3 mL PBS filled falcon tubes and left upright for 60–90 min before inverted until cells float off. A rocker was not required for this method.

### Histopathology

Mucus preparations taken from feces were fixed in formalin and embedded in paraffin. Paraffin sections of 1–2 μm thickness were cut, dewaxed and stained histochemically with hematoxylin and eosin (H&E) for overview and with periodic acid-Schiff (PAS) for mucus. Sections were cover slipped with corbit balsam (Hecht, Germany). For immunohistochemical detection of epithelial cells, paraffin sections were dewaxed and subjected to a heat-induced epitope retrieval step prior to incubation with anti-EpCAM (clone E6V8Y, Cell Signaling). For detection, EnVision+ System- HRP Labeled Polymer Anti-Rabbit (Agilent Technologies) was employed. Nuclei were counterstained with hematoxylin (Merck). Negative controls were performed by omitting the primary antibody. Images were acquired using the AxioImager Z1 microscope (Carl Zeiss MicroImaging, Inc.). All evaluations were performed in a blinded manner.

### Realtime PCR

RNA was isolated from exfoliated cells, intestinal tissue sections and whole fecal pellets that were previously stored at −80°C via homogenization in RNA lysis buffer. The homogenized exfoliated cells, fecal pellets and tissue samples were centrifuged and supernatants were treated with a innuPREP RNA kit (Analytik Jena, Jena, Germany) following manufacturer's instructions. 2 μg of RNA was reverse transcribed to cDNA using a High Capacity RNA to cDNA kit (Applied Biosystems, Foster City, CA). The relative expression of β-actin, resistin-like molecule-beta (RELM-β), IL-25, IL-33, and TSLP was determined by Real Time PCR using 10 ng of cDNA and the FastStart Universal SYBR Green Master Mix (Roche, Basel, Switzerland). Relative gene expression of two-three technical replicates is shown as mean. Primer pairs used for gene amplification were as follows: β-actin (*Actb*) forward: GGCTGTATTCCCCTCCATCG, reverse: CCAGTTGGTAACAATGCCATGT, Relm-β (*Retnlb*) forward: GGCTGTGGATCGTGGGATAT, reverse: GAGGCCCAGTCCATGACTGA, IL-33 (*Il33*) forward: AGGAAGAGATCCTTGCTTGGCAGT, reverse: ACCATCAGCTTCTTCCCATCCACA, IL-25 (*Il25*) forward: ACAGGGACTTGAATCGGGTC, reverse: TGGTAAAGTGGGACGGAGTTG, TSLP (*Tslp*) forward: TGGTAAAGTGGGACGGAGTTG and reverse: TGTGCCATTTCCTGAGTACCGTCA. GAPDH (*Gapdh*) forward: TGGATTTGGACGCATTGGTC, reverse: TTTGCACTGGTACGTGTTGAT; HPRT (*Hprt*) forward: TCCTCCTCAGACCGCTTTT, reverse: TTTCCAAATCCTCGGCATAATGA; GUS-β (*Gusb*) forward: GCTCGGGGCAAATTCCTTTC, reverse: CTGAGGTAGCACAATGCCCA. The mRNA expression was normalized to the β-actin using the Δ-ct value and calculated by Roche Light Cycler 480 software.

### Cycling Conditions

Samples initially undergo denaturation at 95°C for 10 min. The samples are then amplified for 40–50 cycles at a denaturing temperature of 95°C for 15 s. The annealing temperature used was 60°C for 30 s and an elongation temperature of 72°C for 20 s.

### Cytokine Detection by ELISA

RELM-β protein was analyzed using Mouse Resistin-like beta(RETNLB) ELISA kit (CUSABIO, China) according to the manufacturer's instructions. A total of four fecal pellets were pooled from four individual mice and homogenized in 1.2 ml PBS. Samples were then centrifuged at 16,000 g for 5 min. Supernatant was collected and used undiluted for RELM-β detection.

### Fecal Egg Counts

Fecal pellets were collected and placed in glass tubes. Fecal pellets were homogenized and mixed with 1 mL tap water. Next, 6 mL of a saturated salt solution (NaCl) was added to the homogenized sample. The McMaster chamber was then filled with sample and eggs were counted.

### Flow Cytometry

For surface and intracellular staining, the following monoclonal antibodies from BioLegend/eBioscience, San Diego, CA, USA were used:

**Table:** 

**Antibody/clone**	**Flurochrome**	**Vendor**	**Host species**	**Reactivity**
CD4 (RM4-5)	PerCP	BioLegend (Biozol)	Rat	Mouse
IL-13 (eBio13A)	Alexa 488	eBioscience	Rat	Mouse
Foxp3 (FJK-16s)	Alexa 488	eBioscience	Rat	Bovine, Dog, Cat, Mouse, Pig, Rat
GATA-3 (TWAJ)	eFluor 660	eBioscience	Rat	Human, Mouse, Pig, Rhesus monkey
Dead Cell Exclusion Marker	efluor 780	eBioscience		

### Statistics

Experiments are displayed as either mean ± SD or mean ± SEM as indicated. Statistical analysis was completed using GraphPad Prism software (La Jolla, CA, USA). Significance was determined as indicated using the Kruskal-Wallis with Dunn's multiple comparison test or the Mann Whitney *U* Test. Also, multiple t tests were corrected for multiple comparisons using the Holm-Sidak method. Significance was measured as ^*^P ≤ 0.05, ^**^P ≤ 0.01, and ^***^P ≤ 0.001. Data from wild mice was analyzed in R (29) using linear regression. RELM-β expression as a response variable was modeled using the function “lm” with either a) presence of helminths or b) helminth species richness as predictors. For both predictors either i) *Eimeria* oocyst per gram feces, ii) intensity of *Eimeria* tissue stages (as ΔCt Mouse-Eimeria) or iii) presence of *Eimeria*, as determined by both methods, was included as additional predictor variable, allowing for interaction effects. The resulting six models were compared using the Akaike information criterion (AIC) and considered to possess equal explanatory power if ΔAIC < 2.

## Results

### Identification of Epithelial Cells and Mucus in a Layer of Exfoliated Intestinal Cells From Stool

**Figure 1:**
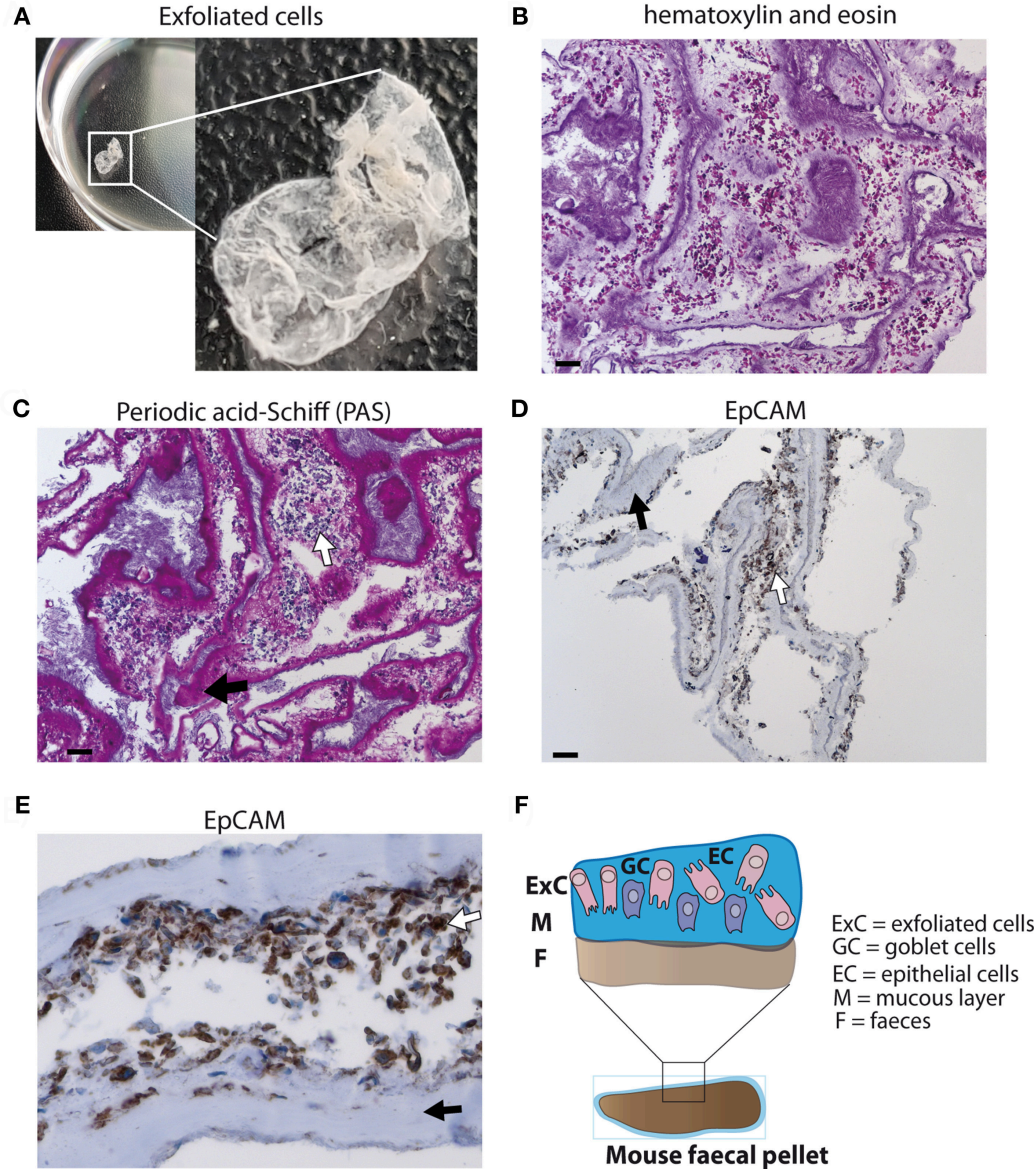
Analysis of exfoliated intestinal epithelial cells isolated from murine stool. **(A)** A layer of exfoliated cells removed from the surface of mouse stool in PBS. **(B)** Hematoxylin and eosin (H&E) stain of exfoliated cells extracted from the surface of stool at 100x magnification (scale bar 100 μm). **(C)** Periodic acid-Schiff (PAS) staining for mucus detection in exfoliated cells at 100x magnification (scale bar 100 μm). **(D)** EpCAM staining identifying epithelial cells using exfoliated cells (brown staining) at 100x magnification (scale bar 100 μm) **(E)** EpCAM staining of epithelial cells at 400x magnification. **(F)** Schematic diagram of stool surface with exfoliated cells. **(C–E)** White arrows display cells and black arrows highlight mucus.

A layer of exfoliated cells from stool (Figure 1A) was analyzed using different histological techniques. A thick mucus layer is apparent in an H&E stain (Figure 1B) and in a PAS-staining (Figure 1C) with cells either attached to the mucus or in between mucinous strands. These cells displayed a broad cytoplasm suggesting that they are of epithelial origin and were probably scaled off during defecation. Immunohistochemical detection of EpCAM expression confirmed that the majority of these cells are epithelial cells (Figures 1D,E). Thus, epithelial cells can be retrieved from the surface of stool (Figure 1F).

### Quantification of RNA Isolated From Exfoliated Intestinal Cells

Currently there is a lack of information on cellular and immunological changes of the gut barrier after infection using non-invasive techniques. Here, we established a method allowing the quantification of RNA from exfoliated intestinal cells. Exfoliated cells showed a lower Cycle threshold (Ct) value for the amplification of RELM-ß compared to homogenized whole fecal pellets. The Ct value is defined as the required number of cycles for the signal to exceed the fluorescence threshold in quantitative PCR (Supplementary Table 1). Furthermore, exfoliated cells showed very low Ct value variation between technical triplicates, whereas homogenized fecal pellets sometimes displayed a difference of 10 in Ct values within triplicates. We attributed the lower quality of the homogenized samples to higher degradation of mRNA based on the differences in Ct values within triplicates. Therefore, we regard the extraction of exfoliated cells to be superior to whole fecal pellet homogenization. To identify the ideal housekeeping gene, the different housekeeping genes β-actin, GAPDH, HPRT and GUS-β were analyzed. β-actin, GAPDH, GUS-β and HPRT were tested using 10 ng cDNA and 100 ng cDNA (Supplementary Table 2). While expression levels of all four housekeeping genes correlated with each other, β-actin produced a lower Ct value at 10 ng compared to the other three housekeeping genes at 100 ng. Thus, β-actin was the housekeeping gene of choice throughout. β-actin was also detectable using fecal samples stored at −20°C in RNA later for 30 days (data not shown).

### RELM-β Expression in Exfoliated Cells During Acute and Chronic Intestinal Helminth Infection

**Figure 2:**
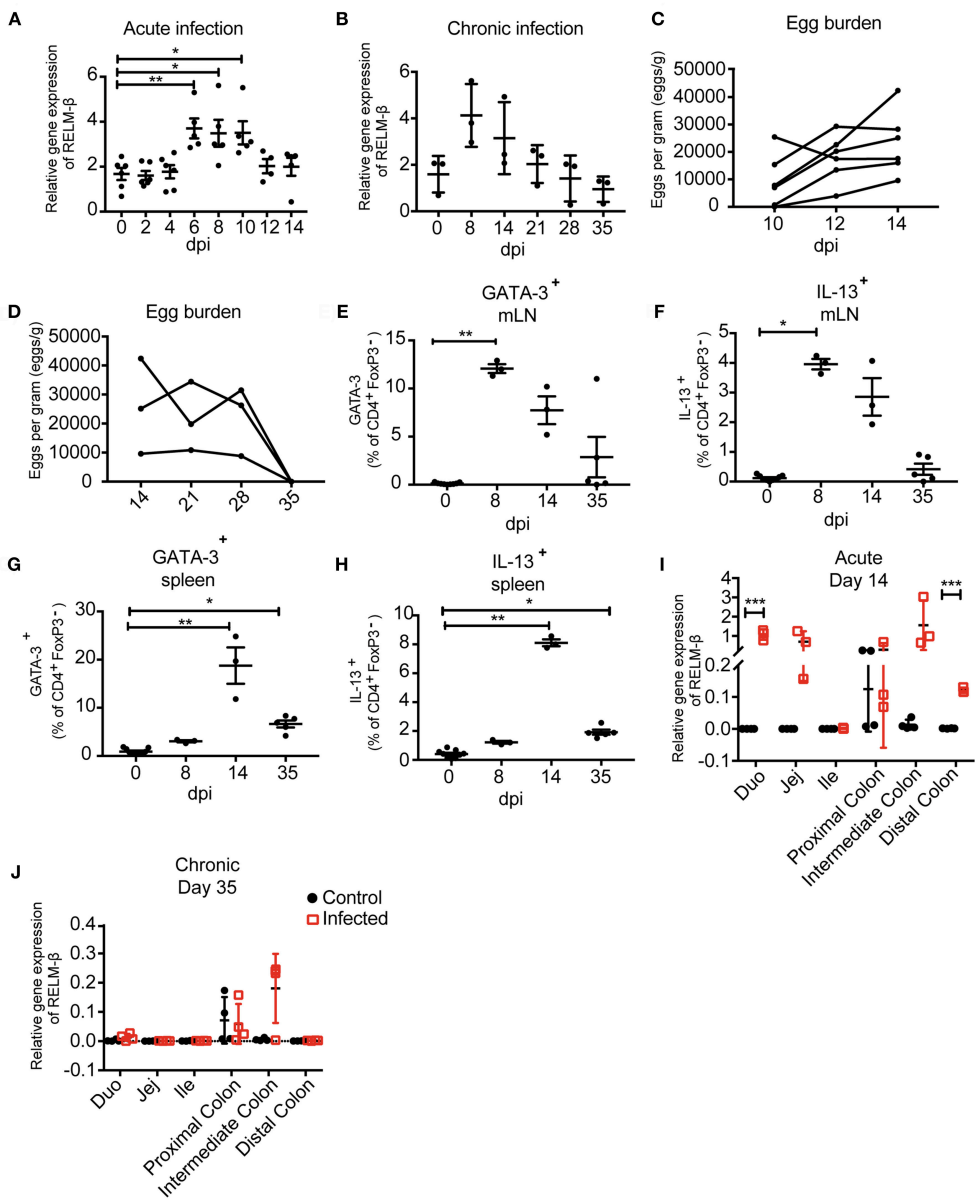
Immunological and parasitological analysis of murine stool after infection with *Heligmosomoides polygyrus*. BALB/c mice were infected orally with 200 infectious L3 stage larvae of *H. polygyrus*. Parameters were measured at different time points and in different regions of the intestine. **(A)** Relative gene expression of RELM-β in exfoliated intestinal cells during acute infection (day 0−14 dpi). **(B)** RELM-β gene expression in exfoliated cells until chronicity of infection (day 0−35 dpi), shown as mean ± SD. **(C)** Fecal egg counts during acute *H. polygyrus* infection (day 14 dpi). **(D)** Fecal egg counts during chronic *H. polygyrus* infection (day 14−35 dpi). Frequency of CD4+ T cells expressing GATA-3 **(E)** and IL-13 **(F)** in mesenteric lymph nodes (mLN). **(G)** Frequencies of CD4^+^ T cell expressing GATA-3 and IL13 **(H)** in spleen. **(I,J)** RELM-β expression in intestinal tissue (duodenum, jejunum, ileum, proximal colon, intermediate colon, and distal colon) at 14 and 35 dpi, respectively. All relative gene expression analysis is compared to β-actin using 10 ng cDNA. Data from **(A)** is pooled from two independent experiments with *n* = 4–10 fecal pellets from 4–6 animals. **(B)** is representative of two independent experiments, *n* = 3. **(C)** is pooled from two independent experiments, *n* = 6. **(D–H)** is representative of two independent experiments, *n* = 3. **(I,J)** is representative of two independent experiments, *n* = 3–4. **(A)** shown as mean ± SEM, ^*^P ≤ 0.05, ^**^P ≤ 0.01, and ^***^P ≤ 0.001. **(B–H)** shown as mean ± SD; **(A–H)** Statistical analysis was performed using the Kruskal-Wallis with Dunn's multiple comparison test, **(I,J)** multiple *t* tests and corrected for multiple comparisons using the Holm-Sidak method, ^*^P ≤ 0.05, ^**^P ≤ 0.01, and ^***^P ≤ 0.001. dpi: days post infection.

Depending on the infection, the immune responses vary in terms of quantity and quality. Here, we aimed to quantify mRNA expression of a selection of immune genes typical for a Th2 type response against helminth infections. We aimed to obtain infection-specific data using exfoliated intestinal cells isolated from stool. To establish this technique, a murine infection model with the small intestinal nematode *H. polygyrus* was used. During the early acute phase of *H. polygyrus* infection the larvae enter the intestinal tissue and develop into the fourth larval (L4) stage, then re-enter the lumen of the gut. In response to the tissue invasive phase and resulting tissue damage the epithelium-derived cytokines IL-25, IL-33, and TSLP are released (31). During an acute *H. polygyrus* infection (14 dpi), our data shows that mRNA expression of these tissue-derived cytokines from exfoliated cells were insufficiently detected and showed high variability between triplicates (Supplementary Figures 1A-C). We thus investigated whether exfoliated cells could be used to detect the Th2 cysteine-rich cytokine RELM-β. RELM-β is produced by intestinal goblet cells that significantly increase during intestinal helminth infection (32). Interestingly, RELM-β expression was significantly detected as early as day 6 post *H. polygyrus* infection (Figure 2A). This increase of expression was also significant at day 8 and 10, steadily decreasing thereafter, making it detectable throughout the course of an acute infection. In contrast, protein detection of RELM-β in feces by ELISA detected an increase in RELM-β at day 8–10 (Supplementary Figure 1G). In the case of chronic helminth infection, RELM-β expression continued to decrease from day 21 to levels similar to day 0 (Figure 2B). As a method to confirm infection, helminth eggs were counted in parallel during the acute and chronic phase (Figures 2C,D). As expected, egg counts and RELM-β mRNA expression peaked at different time points but overall mirrored a similar course of infection. Of note, the RELM-β detection in exfoliated cells directly correlated with the local gut Th2 immune response investigated via flow cytometry during the course of infection. An increase in the Th2 transcription factor GATA-3^+^ in CD4^+^ T cells in mesenteric lymph nodes (mLN) peaked at day 8 post *H. polygyrus* infection (Figure 2E). The same was observed for the Th2 cytokine IL-13 (Figure 2F). When investigating the systemic immune response, we observed a delayed peak in the expression of GATA-3^+^ in CD4^+^ T cells at day 14 in the spleen, decreasing onwards to day 35 (Figure 2G). The same was observed for the IL-13 expression in the spleen indicating a delayed systemic response to infection (Figure 2H). Thus, the local immune response in the infection draining mLN mirrored RELM-β detection in exfoliated cells.

To verify the observed mRNA levels in exfoliated cells, we examined the cytokine expression in host tissue along the gut. In parallel, intestinal tissues from the duodenum, jejunum, ileum, proximal colon, intermediate colon and distal colon were analyzed to decipher the expression of the tissue derived cytokines IL-25, IL-33, TSLP (Supplementary Figures 1D-F) and the goblet cell produced RELM-β. Interestingly, at 14 days post-infection a significant RELM-β signal was detectable in the duodenum and distal colon during acute *H. polygyrus* infection (Figure 2I). However, RELM-β mRNA was not observed in intestinal tissue at day 35 post infection (Figure 2J) correlating with the RELM-β detection in exfoliated cells. This data indicates a reduced Th2 immune response against the worm infection, probably due to advanced clearance of the parasite.

In summary, RELM-β mRNA as an indicator for gut barrier changes was detectable as early as day 6 post nematode infection in exfoliated intestinal cells. The non-invasively detected RELM- β expression mirrored the immune responses at the gut barrier. Thus, exfoliated epithelial cells from stool can be used to detect infection.

### Pathogen Dose-Dependent Expression of the Cysteine-Rich Cytokine RELM-β in Exfoliated Epithelial Cells

**Figure 3:**
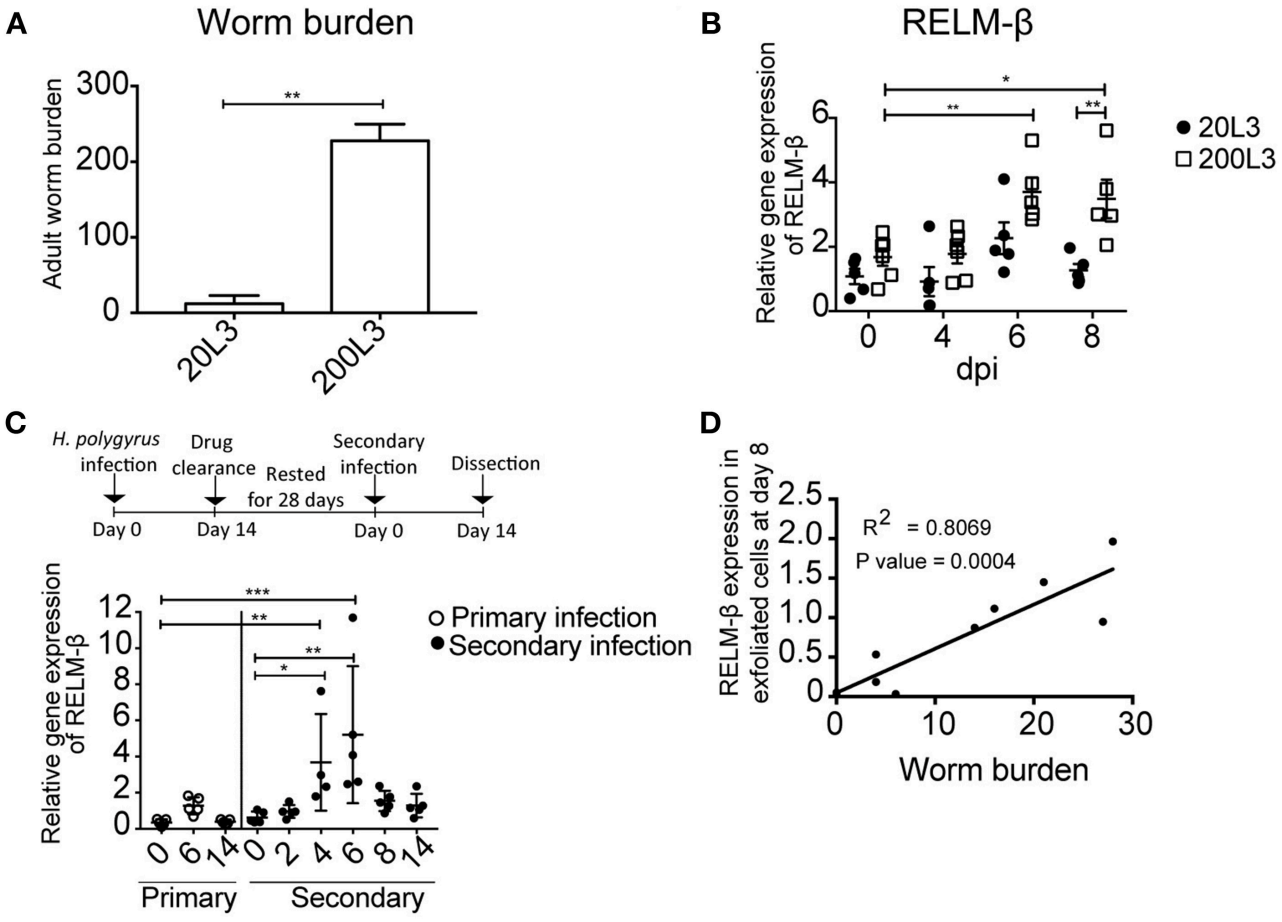
RELM-β expression in exfoliated cells reflects the intensity and type of infection. BALB/c mice were infected orally with either a low dose (20 L3) or a high dose (200 L3) *H. polygyrus* larvae. **(A)** Worm burden assessed at 14 dpi. **(B)** RELM-β relative gene expression during early *H. polygyrus* infection until day 8 dpi after low dose (20 L3) and high dose (200 L3) infection. **(C)** BALB/c mice were infected orally with 200 L3 *H. polygyrus*, treated with pyrantel pamoate for worm clearance and 28 days later reinfected with 200 L3. RELM-β gene expression shown during primary and secondary infection. **(D)** Linear regression comparing RELM-β gene expression at day 8 in exfoliated cells with low worm burden. **(A)** is representative from two independent experiments with *n* = 3–5 animals and shows the mean ± SD. **(B)** is pooled from two independent experiments with *n* = 4–6 animals, data shown as mean ± SEM. All relative gene expression analysis is compared to β-actin using 10ng cDNA. **(C)** is representative of two independent experiments, *n* = 4–5. **(D)** is pooled from two independent experiments, *n* = 10. Statistical analysis using **(A)** Mann Whitney U Test, **(B)** multiple *t* tests and corrected for multiple comparisons using the Holm-Sidak method, **(C)** was performed using the Kruskal-Wallis with Dunn's multiple comparison test. All relative gene expression analysis is compared to β-actin. Significance was measured as ^*^P ≤ 0.05, ^**^P ≤ 0.01, and ^***^P ≤ 0.001.

Next, we aimed to investigate if the detection of the cysteine-rich cytokine RELM-β correlated to the intensity of infection. Changes in the gene expression of RELM-β in exfoliated gut cells were studied using different infection dosages. Mice were infected with either a low dose infection with 20 L3 of *H. polygyrus* or a high dose infection with 200 L3 and adult worm burden was assessed at day 14 (Figure 3A). The high dose infection was reflected by a higher RELM-β expression compared to a low dose infection. The expression of RELM-β during the 200 L3 infection was significantly higher compared to a low dose infection at day 8 p.i. (Figure 3B). This effect was most prominent at day 8 but a trend was detected from day 4 p.i. onwards. Thus, RELM-β expression in exfoliated cells does mimic the intensity of an intestinal infection.

In nature, animals frequently get re-infected with helminths. Therefore, we asked if our new non-invasive method is suitable to detect the expression of RELM-β as a marker for gut barrier changes after drug clearance of *H. polygyrus* and reinfection. Mice were re-infected 28 days after drug clearance with 200 L3 infective larvae. RELM-β mRNA was significantly detectable in exfoliated gut cells at day 4 and 6 in mice re-infected with *H. polygyrus* (Figure 3C). Interestingly, a significantly positive linear relationship was observed between the expression of RELM-β in exfoliated cells and adult worm counts (Figure 3D).

### Analysis of Intestinal Exfoliated Cell Gene Expression in Wild Mice Samples

**Figure 4:**
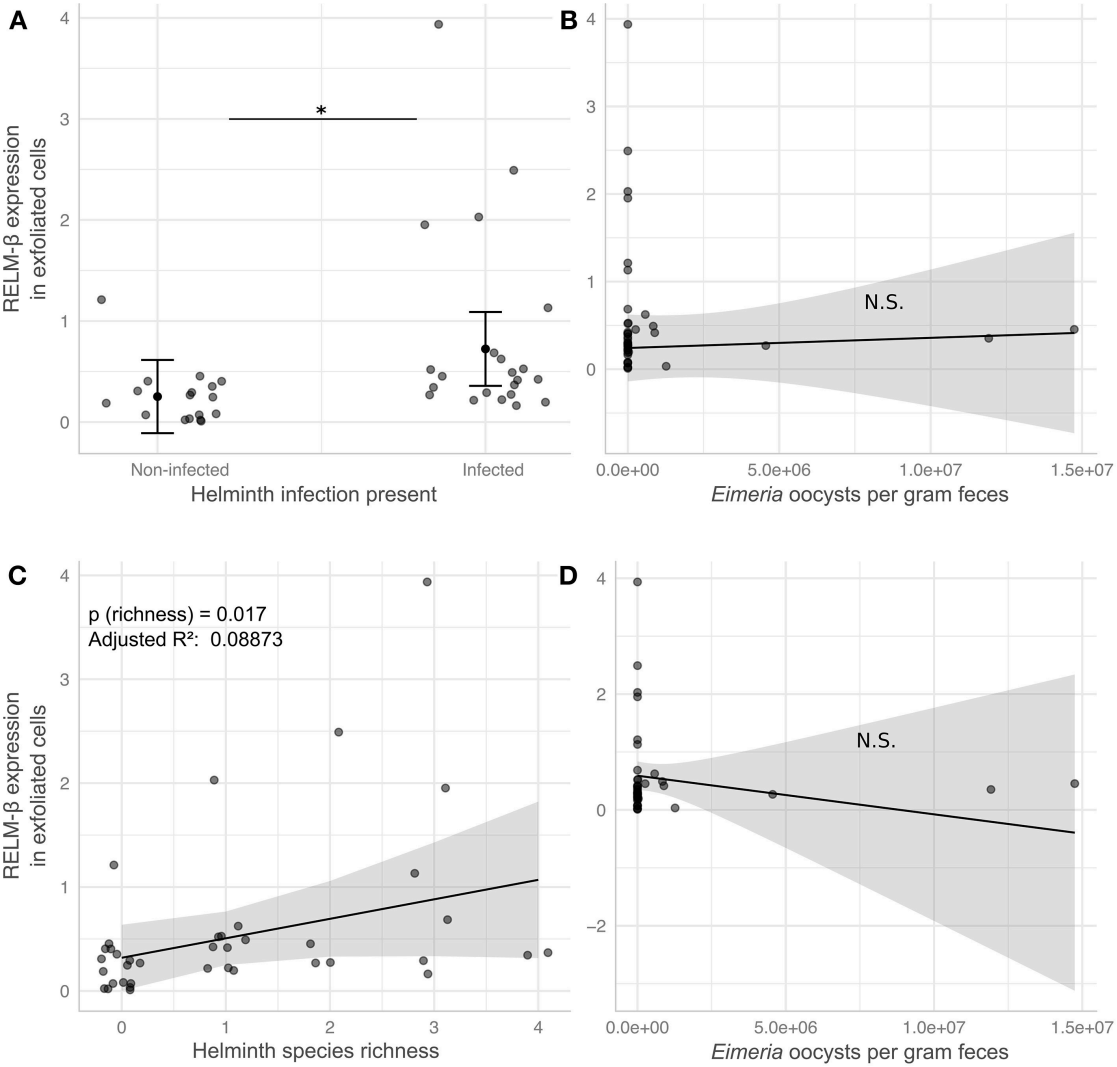
RELM-β expression in exfoliated cells differs in response to helminth infection in wild house mice. Helminth and *Eimeria* infections were assessed in wild house mice. Linear models predict RELM-β expression to be significantly elevated by either presence **(A)** or species richness **(C)** of helminths, while the number of *Eimeria* oocysts shed per gram feces in the same mice has no significant effect in the respective models for presence **(B)** and richness **(D)**. Gray points depict values for individual mice and are jittered relative to the x-axis to avoid overplotting in **(A)**, solid black points and lines indicate marginal means estimated in statistical models (see Supplementary Table 4; Model 3 and Model 6), error bars and shaded areas represent 95% confidence intervals for these estimates. ^*^P ≤ 0.05.

In order to analyze the potential of the non-invasive cytokine detection method with wildlife animals, we tested stool samples collected from wild *Mus musculus* mice captured in Brandenburg, Germany. RELM-β gene expression was analyzed in exfoliated intestinal cells isolated from fecal pellets from wild mice with no worms detectable in the gut. The data was then compared to data obtained from wild mice with natural infections of eight species of helminths (Supplementary Table 3). We observed a significant increase of RELM-β expression in samples from wild mice in the presence of worms and with species richness of helminths in comparison to mice without helminths. In addition, we studied the effect of a coinfection with the protozoan gut parasite *Eimeria* spp. on the expression of RELM-β in helminth-infected mice vs. mice without helminths. We scrutinized these results using six statistical models in total (Supplementary Table 4). All models found a significant increase of RELM-β expression in the presence of the gastrointestinal helminths (Figure 4A). This was independent of *Eimeria* spp. load in the tissues and *Eimeria* spp. oocyst abundance in the feces (Figures 4A,B) or the presence of *Eimeria* as a second predictor. The statistical models predicted a significant increase in RELM-β expression with helminth richness (Figure 4C) when controlling for *Eimeria* spp. tissue load or oocyst abundance (Figures 4C,D). Furthermore, a trend for higher RELM-β expression and species richness was determined when including *Eimeria* presence in the model. All the models had similar quality as assessed by the Akaike information criterion (AIC). Models predicting RELM-β expression with the abundance of individual worm species did not show significant differences (data not shown).

In conclusion, our study with samples from wild animals shows that RELM-β is induced during an active infection with intestinal helminths and this expression was robust in presence of a coinfection with the gut protozoan parasite *Eimeria spp*.

## Discussion

Here we investigated whether exfoliated intestinal cells released with feces mirror immunological host responses corresponding to gut barrier changes during a gastrointestinal nematode infection with *H. polygyrus*. Investigation into infection related genes revealed the goblet cell-secreted RELM-β as a reliable marker reporting gut barrier changes due to acute worm infection. RELM-β was significantly detectable during the early stages of an acute *H. polygyrus* infection and decreased steadily thereafter. Intriguingly the mRNA levels of RELM-β in exfoliated cells reflected the local GATA-3 and IL-13 expression in mLN during infection. Our data suggests that detection of RELM-β in exfoliated gut cells not only detects gut barrier related changes but also reflects the local Th2 immune response.

In the intestine, epithelial cells represent an essential barrier that undergoes continuous homeostatic turnover replacing old cells and lost or damaged epithelial cells during infection (33, 34). Additionally, epithelial cells are part of the mucosal immune response and aid in eliciting the prominent Th2 immune response during gastrointestinal helminth infection. Exfoliated cells are shed daily in feces and have been previously described to be of colonic origin (35). Interestingly during *H. polygyrus* infection goblet cells (GCs) play an important role in the secretion of RELM-β. The secretion of this cysteine-rich cytokine in intestinal tissue is essential for normal spontaneous worm expulsion (12). RELM-β acts on *H. polygyrus* by impairing feeding thus reducing protein and ATP content, leading to pathogen expulsion (31). Goblet cells also secrete mucin forming a mucosal surface barrier limiting interactions with luminal microbes (36). Additionally, during homeostasis GCs are highly abundant in the colon compared to the upper intestinal tract (13).

Importantly, our method is applicable in the field where cooling and proper storage of biological material is limited. We tested fecal pellets from wild mice freshly placed in RNA later™ and then stored at −20°C for 30 days. Interestingly, β-actin expression was detectable with a Ct value of 25, similar to flash frozen and −80°C stored fecal pellets (data not shown). Thus, this non-invasive method allows stool to be stored for longer periods of time before analysis, whereas egg counts need to be processed as soon as possible for a reliable representation of infection (37). This suggests that both egg counts and exfoliated cells should be used together to complement the information not only on the infection status but in addition the cellular host responses at the gut barrier.

Nematodes dwelling in the small intestine, such as *H. polygyrus* have been shown to affect colonic permeability (38). This cellular response in the colon to an infection dwelling in the small intestine enables the detection of cellular responses using exfoliated cells likely originating from the colon. In addition, gastrointestinal nematodes have been described to alter the gut bacterial environment (13, 39). Increase in abundance of gram-negative bacteria during *H. polygyrus* infection (13, 14), accompanied by colonic barrier permeability (38) correlate with the increased early detection of RELM-β during infection in our study. Here, it is interesting to note that RELM-β is highly expressed (34) and plays multiple roles, such as recruiting CD4^+^ T cells in *Citrobacter rodentium* infection (10). Additionally, RELM-β has been described as a bactericidal protein that kills gram-negative bacteria, limiting the association of bacteria with colonic tissues (11). Thus, the ability to detect RELM-β in exfoliated cells can possibly be attributed to the highly abundant goblet cells in the large intestine secreting RELM-β, to deal with gut barrier changes and the increase in bacteria during nematode infection. Thus, the previously described altered colonic permeability (38) and the increased gram-negative bacteria (13, 14) explain the high expression of RELM-β and the detectability in exfoliated cells compared to the tissue cytokines.

Comparison of the tissue cytokines in different tissue compartments at 14 dpi confirmed no significant changes in IL-25, IL-33 and TSLP in the distal colon (Supplementary Figures 1A-C). Additionally, expression of these tissue cytokines could not be detected using exfoliated cells from stool. The observed systemic tissue RELM-β expression and high expression in exfoliated cells displays a reliable marker that mirrors the local *H. polygyrus* Th2 immune responses non-invasively. Additionally, this method can be utilized for other colonic infections and might display RELM-β as a marker of gut barrier changes.

In order to identify if exfoliated cells can be used as a measure of infection load, we tested a low vs. high dose infection and identified significant differences in RELM-β expression. This method detected a difference in infection dose highlighting its potential as a marker of infection load and also reported the quality of the immune response in a secondary infection. A linear regression comparison of exfoliated cells compared to worm burden revealed a significantly positive relationship. Interestingly, RELM-β expression in exfoliated cells mirrored the kinetics of the local immune response in our study. Consequently, detection of RELM-β in exfoliated cells might be useful not only as an additional measure to egg counts but also as a means to monitor cellular gut barrier changes correlating to infection loads. However, previous studies have shown that RELM-β is detectable on the protein level in feces via western blot (10) and ELISA (Supplementary Figure 1G). On the other hand, our method described here investigates transcript levels using exfoliated cells. This is especially interesting as the ELISA is dependent on the availability of species-specific antibodies against the gene of interest. Thus, the lack of antibodies for certain genes restricts the potential use of protein analysis. Antibodies are also restricted to specific animal species making it difficult to detect certain proteins. Therefore, this method allows for a cost effective alternative to protein studies by ELISA.

We believe that our novel method of using exfoliated cells to detect changes at the gut barrier might pave the way for further investigation into infection-specific markers under laboratory settings but also in infections of wild life animals.

In wildlife, animals are exposed to a variety of pathogens and monitoring of the health status is urgently needed for wildlife conservation, zoonotic diseases and disease control. However, the only methods applicable to wildlife studies are non-invasive methods. Over time there have been many advances in different molecular biology techniques allowing for in depth research into different diseases. However, not much is known regarding cellular changes during infection in natural animal populations. We demonstrate here that our method using exfoliated cells to investigate infection related gut barrier changes works in both laboratory and wild animals. We found RELM-β to be a marker for helminth infection in wild house mice without being able to pinpoint a single worm species as most relevant driver of the RELM-β expression. A significant effect of worm species richness on RELM-β expression could indicate that the marker might be suitable to detect general stress on the gut barrier in wild systems. This prospect warrants further investigation.

*Eimeria* spp. is the most prevalent protozoan parasite in the studied house mice (Jarquin et al. unpublished) and could have a potential effect on RELM-β expression, such as inducing an opposing immune response. We were able to test the influence of *Eimeria* spp. on RELM-β expression, including co-infections with worms, but did not find an effect despite investigating a sufficient sample size. We thus conclude that there is no indication of protozoan infections affecting RELM-β expression at the gut barrier in helminth-infected wild mice hosts.

In conclusion, we describe a novel method to measure cellular parameters using exfoliated cells from stool to detect infection-related gut barrier changes in samples from wildlife and experimental laboratory conditions. This study paves the way for further investigation into infection-specific markers under laboratory settings but also in infections of wild animals. Further investigation into pathogen-specific infection markers is required. Thus far, our technique represents a potential addition to lab and field non-invasive techniques reflecting gut barrier changes. RELM-β detection in exfoliated cells can potentially provide a suitable marker for colonic inflammation in a variety of different diseases and colitis infections.

## Data Availability

All datasets generated for this study are included in the manuscript and/or the Supplementary Files.

## Author Contributions

NoA and NX performed all the exfoliated cell extractions and gene expression experiments. SH and SS conceptualized and designed the research. NiA performed all the flow cytometry experiments and analysis. EH provided input into all wild mouse experimental data and analysis. AK performed all the histopathology experiments. VJ was involved in the capturing and sample collection from wild mice and JJ was involved in the identification of worms in wild mouse samples. NoA and SH wrote the manuscript. All authors approved the final version of the manuscript.

### Conflict of Interest Statement

The authors declare that the research was conducted in the absence of any commercial or financial relationships that could be construed as a potential conflict of interest.

## Supplementary Material

Supplementary Figure 1 BALB/c mice were infected orally with 200 infectious L3 stage larvae of *H. polygyrus*. Parameters were measured at different time points and in different regions of the intestine. **(A)** Relative gene expression of IL25, **(B)** IL33 **(C)** TSLP in exfoliated intestinal cells during acute infection (day 0–14 dpi). Relative gene expression of **(D)** IL25, **(E)** IL33, **(F)** TSLP in intestinal tissue (duodenum, jejunum, ileum, proximal colon, intermediate colon, and distal colon) at 14 dpi. **(G)** RELM-β protein detection via ELISA using undiluted fecal supernatant.

Supplementary Table 1 Real Time PCR cycle threshold (Ct) values of β-actin in exfoliated cells and homogenized stool using a 10 and 100 ng cDNA concentrations.

Supplementary Table 2 Exfoliated cells were used to detect the Ct values of the different housekeeping genes β-actin, GAPDH, HPRT and GUS-β using 10 and 100 ng cDNA concentrations.

Supplementary Table 3 Represents the location, sex, and infection status of wild *Mus musculus* mice captured in Brandenburg, Germany.

Supplementary Table 4 Represents the different statistical models used for wild *Mus musculus* mice captured in Brandenburg, Germany.

Supplementary Video 1 Shows exfoliated cells being removed from whole fecal pellets.
