# Supplementary figures and images for: A Novel Non-invasive Method to Detect RELM Beta Transcript in Gut Barrier Related Changes During a Gastrointestinal Nematode Infection
Source: Front Immunol. 2019 Mar 12;10:445. doi: 10.3389/fimmu.2019.00445 (PMC6423163; doi:10.3389/fimmu.2019.00445)

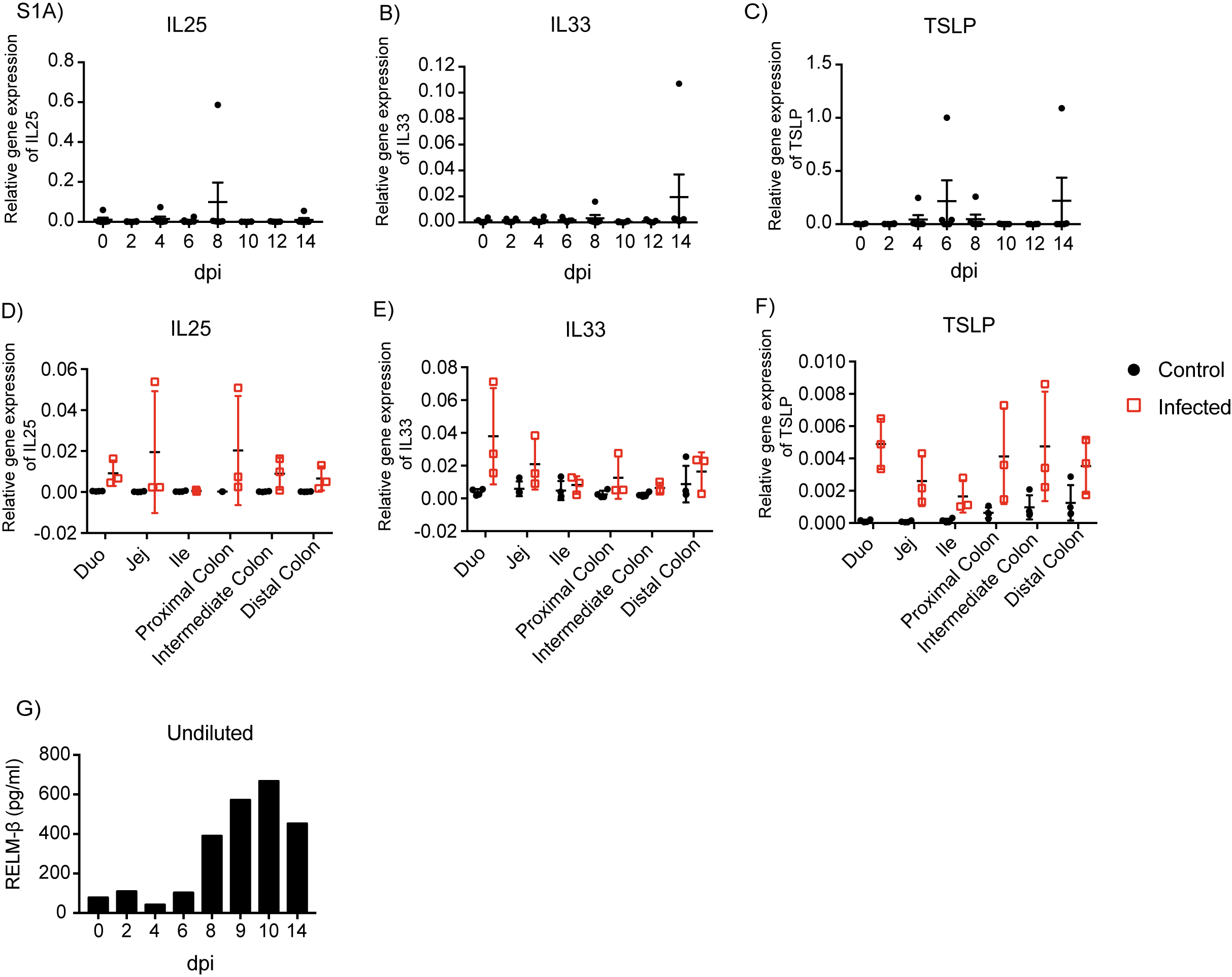

Supplement: Supplementary Figure 1 — BALB/c mice were infected orally with 200 infectious L3 stage larvae of H. polygyrus. Parameters were measured at different time points and in different regions of the intestine. (A) Relative gene expression of IL25, (B) IL33 (C) TSLP in exfoliated intestinal cells during acute infection (day 0–14 dpi). Relative gene expression of (D) IL25, (E) IL33, (F) TSLP in intestinal tissue (duodenum, jejunum, ileum, proximal colon, intermediate colon, and distal colon) at 14 dpi. (G) RELM-β protein detection via ELISA using undiluted fecal supernatant. [file Image_1.TIF]
